# Influence of Gallic Acid-Containing Mouth Spray on Dental Health and Oral Microbiota of Healthy Dogs: A Pilot Study

**DOI:** 10.3390/vetsci10070424

**Published:** 2023-06-30

**Authors:** Nichaphat Thongma, Bhagavathi Sundaram Sivamaruthi, Muruganantham Bharathi, Chawin Tansrisook, Sartjin Peerajan, Kittidaj Tanongpitchayes, Natcha Chawnan, Subramanian Rashmi, Kriangkrai Thongkorn, Chaiyavat Chaiyasut

**Affiliations:** 1 Innovation Center for Holistic Health, Nutraceuticals and Cosmeceuticals, Faculty of Pharmacy, Chiang Mai University, Chiang Mai 50200, Thailandsivamaruthi.b@cmu.ac.th (B.S.S.); kittidaj_tanong@cmu.ac.th (K.T.);; 2 Small Animal Hospital, Faculty of Veterinary Medicine, Chiang Mai University, Chiang Mai 50200, Thailand; 3 Office of Research Administration, Chiang Mai University, Chiang Mai 50200, Thailand; 4 Health Innovation Institute, Chiangmai 50200, Thailand; 5 Department of Companion Animal and Wildlife Clinic, Faculty of Veterinary Medicine, Chiang Mai University, Chiang Mai 50100, Thailand

**Keywords:** gallic acid, oral microbiome, gingival index, plaque index, calculus index, QIIME 2.0^TM^

## Abstract

**Simple Summary:**

Dogs frequently have oral diseases, especially periodontal diseases. Medications for oral health are being used in dogs to prevent infections. Oral microbiota plays a major role in dental health. The present study displayed the effect of gallic acid-containing mouth spray (GAMS) on healthy dogs’ oral health and microbiota. The mouth spray improved the gingival and calculus indexes in healthy dogs. GAMS altered the dental microbiota. Further studies are obligatory to claim that the GAMS could be used to maintain the oral health of healthy dogs.

**Abstract:**

The pilot study aimed to investigate the effects of GAMS on oral microbiota in healthy dog subjects. Thirty-eight dogs were recruited and randomly allocated to the placebo (*n* = 19) and treatment groups (*n* = 19). The dogs were treated with mouth spray once daily for 42 days. The changes in the gingival index (GI), plaque index (PI), and calculus index (CI) were measured at baseline (day 0) and end of the study (42nd day). The changes in the oral microbial composition of representative dogs (placebo, *n* = 7; and treatment, *n* = 7) were also evaluated at baseline and end of the study. Oral microbial composition was assessed by sequencing. The sequences were annotated using the QIIME 2.0^TM^. The GI, PI, and CI indexes were reduced after the GAMS usage. The abundance of the commensal bacterial phylum Actinobacteria and Chloroflexi, genera *Frederiksenia*, and *Bergeyella* was improved after six weeks of GAMS usage. GAMS reduced the pathogenic bacterial species, including *Neisseria* sp., *Desulfobulbus* sp., *Capnocytophaga canis*, and *Corynebacterium mustelae.* Moreover, some pathogenic bacterial abundances were increased at the end of the study. All the microbial variations were observed within the group. The inter-group analysis revealed that the changes were unrelated to GAMS usage. Further studies need to be carried out using more experimental subjects to confirm the effectiveness of GAMS. More metagenomic data are required to evidence the GMAS impact on the oral microbiome of healthy dogs.

## 1. Introduction

The oral cavity is a habitat for several microbial species. Several factors, including food matter, care regimens, and oral care, influence the composition and functional contributions of the microbial population [1]. Extreme microbial dysbiosis promotes the risk of developing periodontal diseases (PD), an inflammatory condition marked by a complicated interplay between the immune function and oral microorganisms. Biofilm forms and multiplies on the tooth surface (supragingival plaque) before spreading under the gingiva (subgingival plaque); in contact with the gingiva, dental plaques are activated, and inflammatory reaction indicates the disease’s progress and may cause the loss of teeth [2]. PD is one of the most common illnesses in dogs globally, affecting over 70% of dog sufferers [3,4,5,6].

The alternation of microbial composition is closely associated with PD development. For example, the abundances of Actinobacteria and Proteobacteria were decreased, and Bacteroidetes, *Porphyromonas*, and *Tannerella* spp. abundances were higher in PD conditions [7].

Professional dental cleaning requires scaling or extraction as the dental calculus and tooth loss condition progresses severely [8,9]. Moreover, professional cleaning was of little value without home care.

Daily dental home care, such as brushing, prevents and controls PD in people and dogs. Even though a manual, an ultrasonic toothbrush, a nylon glove, and a microfiber finger cloth help clean the teeth, daily brushing is difficult [10].

Several chemicals and enzyme-based therapeutic products were reported to manage PD. Chlorhexidine (CHX), a cationic biguanide, could damage the bacterial biofilm and have anti-viral and anti-fungal properties [11]. However, CHX has side effects when administered for a long time, including loss of taste, acceleration of the plaque’s calcification, enamel discoloration, and oral mucosal ulcers [11,12]. In dogs, xylitol-based drinking water was used for dental plaque reduction [13,14]. Few studies reported that consuming foods containing high concentrations of xylitol may cause some adverse effects in dogs [15,16]. Nisin-based mouthwash could decrease canine gingivitis [17], and nisin slightly impacted canine commensal oral microbiota [18].

Glucose oxidase and lactoperoxidase-based oral care products such as rinses and toothpaste also have some limitations, including their sensitivity to proteolysis, low stability in oral hygiene products, and insufficient retention time in the oral cavity [19,20]. The use of therapeutic dental chews in dry dog diets significantly decreased the build-up of plaque and calculus and the severity of their gingivitis [21]. However, chewing hard objects might be problematic because severe abrasion could stimulate the gingival edge to migrate apically and cause tooth fractures [11,22]. Hence, further research is needed to develop potent oral care products for dogs with fewer side effects and be cost-effective and user-friendly.

Gallic acid is a natural phenolic compound of several plants, known for its health-promoting properties [23]. Gallic acid could prevent the adherence and formation of biofilm by several pathogens (*Pseudomonas aeruginosa*, *Listeria monocytogenes*, and *Staphylococcus aureus*), especially oral pathogen (*Streptococcus mutans*) [24,25,26]. Hesperidin methyl chalcone, poloxamer188, and sodium metabisulfite (preservative) are the other ingredients of formulated mouth spray. Hesperidin methyl chalcone has been reported for its anti-inflammatory and antioxidant properties [27,28]. Poloxamer188 has also been reported for its anti-inflammatory property [29,30]. FDA-approved appropriate concentrations of sulfite preservatives are considered as safe. Sulfite preservatives alter the richness and relative abundance of microbiota present in the saliva. Ten minutes of exposure to sulfite preservatives reduce the number of viable cells [31].

Recently, we reported the efficiency of gallic acid-containing mouth spray (GAMS) on cats’ oral health. GAMS improve the gingival and plaque indexes and promote the richness of commensal microbes *Porphyromonas* and *Moraxella*, and act against pathogens, especially *D. orale*, in cats [32]. Since the GAMS was effective in the improvement of oral hygiene of cats [32], the diversity of oral microbiota of felines slightly varies compared to the dogs [33]. If the GAMS was effective against periodontal pathogens, it could be used to improve the oral hygiene of the dogs. Hence, we aimed to study the efficiency of GAMS on dogs’ oral health.

## 2. Materials and Methods

### 2.1. Preparation of GAMS

A detailed composition of the GAMS was reported in our previous study [34]. Briefly, gallic acid, ZnCl_2_, poloxamer188, poloxamer407, carrageenan, hesperidin methyl chalcone, sodium metabisulfite, and water were mixed in the reported ratio [32].

### 2.2. Experimental Animals

Thirty-eight healthy dogs were recruited for the study. The veterinarian confirmed the dogs’ health through physical examination, thoracic radiography, complete blood count (CBC), and blood biochemistry (blood urea nitrogen, creatinine, alanine aminotransferase (ALT), and alkaline phosphatase (ALP)). The mesocephalic skull dogs aged over 2 years with complete investigating teeth (104, 108, 204, 208, 304, 309, 404, and 409) were included in the study. Dogs on medications (steroids, non-steroidal anti-inflammatory drugs, or antibiotics) or allergic to oral spray were excluded from the study. The animal care and use committee, Faculty of Veterinary Medicine, Chiang Mai University, Chiang Mai, Thailand, approved the experiment (Ref. No. R2/2564). Dogs were randomly divided into experimental groups (G1; *n* = 19) and a control group (G2; *n* = 19).

### 2.3. Dental Procedure and Mouth Spray Administration

Before experimentation, a general physical examination was performed for dogs. Amounts of 0.2 mg/kg diazepam (Diazepam^®^; Government Pharmaceutical Organization (GPO), Bangkok, Thailand) (intravenous administration) and 4 mg/kg tramadol (Tramadol^®^; T.P. Drug Laboratories (1969) Co., Ltd., Bangkok, Thailand) (subcutaneous administration) were used for sedation. After sedation, dogs received 4–6 mg/kg propofol (Pofol^®^; Dongkook Pharmaceutical Co., Ltd., Chungcheongbuk-do, Republic of Korea) (intravenous administration) to stimulate anesthesia. An endotracheal tube was then intubated, and the cuff was filled. The anesthesia stage was maintained using isoflurane (Attane^®^; Piramal Critical Care, Inc., Pennsylvania, United States of America) and oxygen during the dental procedure. Periodontal probing, oral evaluation, and dental charting were performed at baseline (week 0) and after 6 weeks of study, and the changes were recorded per the modified Triadan system. The dental indices were measured on (bilaterally) maxilla: canine and fourth premolar (104, 204, 108, 208), and mandible: canine and first molar (304, 404, 309, 409). G1 was treated with mouth spray (1 mL of spray = 10 puffs; Dose: 4 puffs per day) before bed and withheld food and water for half an hour after application for 6 weeks. The control group (G2) was not subjected to any treatment.

### 2.4. Assessment of Plaque, Calculus, and Gingivitis Indexes

The plaque index (PI), calculus index (CI), and gingivitis index (GI) were calculated using the Logan and Boyce index [35] (Table S1), the Warrick and Gorrel method [36] (Table S2), and the Löe and Silness index [37] (Table S3), respectively.

### 2.5. Next-Generation Sequencing (NGS)

NGS evaluated the oral microbiome of the experimental dogs. The saliva samples were collected at baseline and after six weeks of study. QIAamp UCP DNA Micro Kit (QIAGEN, Hilden, Germany) was used to isolate the total genomic DNA from the saliva samples. The sequencing was performed at the Omics Sciences and Bioinformatics Center, Faculty of Science, Chulalongkorn University, Bangkok, Thailand, as described in our previous study [34].

### 2.6. Statistical Analysis

Fisher’s exact, Mann–Whitney U, and Wilcoxon signed-rank tests were performed to determine the experimental subjects’ significant changes in the studied parameters and characteristics. The changes were considered significant if the *p* < 0.05. The values were presented as median ± interquartile ranges. The oral microbial sequences were analyzed using the QIIME2.0^TM^. Alpha diversity was analyzed using the metric pielou_e, Simpson, and Shannon entropy to measure the relative evenness of species richness, abundance, and taxonomical diversity with statistical significance (*p* ≤ 0.05). The beta diversity was measured for the control (Cpre vs. Cpost) and treatment (Tpre vs. Tpost) groups using the unweighted UniFrac distances matrix with a 3D PCoA plot. The raw operational taxonomic units (OTUs) were used to determine phylum to species and compared the phylum in the control and treatment group as a heat map. The relative frequency was calculated using the observed OTUs for the phylum, genera, and species. The taxonomical differences between the Cpre, Cpost, Tpre, and Tpost samples were estimated using the Wilcoxon signed-rank test with a statistical significance of *p* ≤ 0.05.

## 3. Results

### 3.1. Demographic Data and Changes in GI, PI, and CI

The gender, weight, and age of the dogs were not significantly different during the study (Table 1). GI, PI, and CI were measured at baseline and after six weeks of the study. Eight teeth and their three (GI and CI) and two (PI) positions were utilized for the measurements. The results were represented as median and interquartile (IQ) values.

The GI values of the tooth code 104, position 3 (*p* = 0.0148); 108, positions 1 and 2 (*p* = 0.0148 and 0.0459); 204, position 3 (*p* = 0.0009); 208, position 3 (*p* = 0.005); and 409, position 2 and 3 (*p* = 0), were significantly reduced after treatment. The PI value of the tooth code 204, position 1 (*p* = 0.0293), was significantly reduced after treatment. The CI values of tooth code 104 (position 3), 108 (in all positions), 204 (in all positions), 208 (in all positions), 304 (position 2), 309 (position 1 and 2), 404 (position 3), and 409 (position 1 and 3) were significantly reduced after treatment. No significant changes were observed in GI and PI values in the control group, whereas CI values of the tooth codes 108, 204, 208, 404, and 409 were also reduced after 6 weeks in the control group (Table 2).

### 3.2. Oral Microbiome Analysis

The baseline (Cpre) and sixth-week samples (Cpost) had 455,410 and 442,275 microbial sequence reads in the control group, respectively. The non-chimeric sequences of 350,430 and 321,204 were retrieved in Cpre and Cpost samples. Moreover, 343,924 and 351,717 microbial sequence reads were obtained from the treatment group’s baseline (Tpre) and sixth-week samples (Tpost), respectively. The non-chimeric sequences 264,859 and 261,791 were retrieved in the Tpre and Tpost samples for further analysis (Table S4).

#### 3.2.1. Alpha Diversity

**Figure 1:**
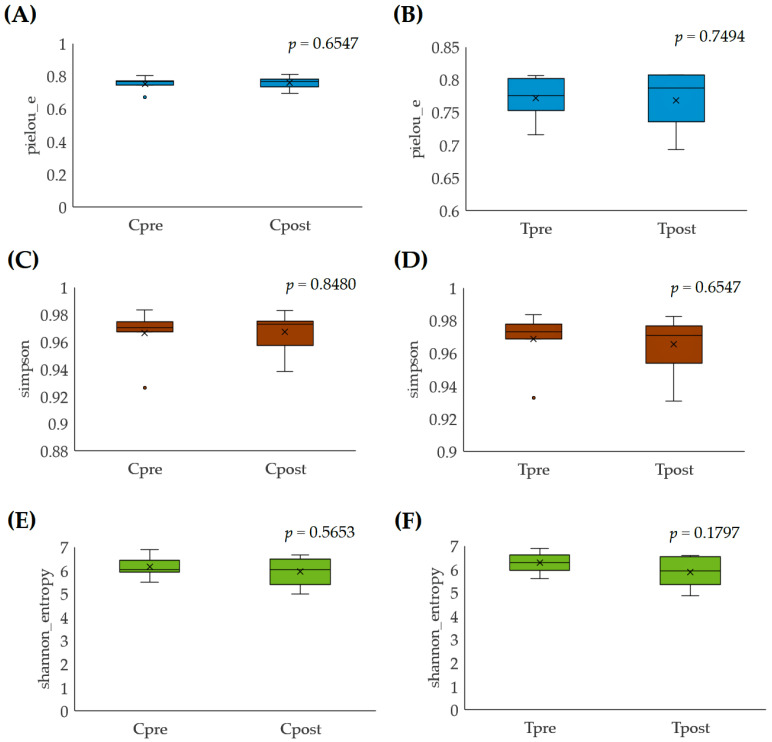
Alpha-rarefaction analysis for the control and treatment group samples: (**A**,**B**) pielou_e; (**C**,**D**) Simpson; (**E**,**F**) Shannon entropy.

The alpha diversity was estimated by Pielou’s evenness, Simpson index, and Shannon index with the Kruskal–Wallis (pairwise) test. Pielou’s evenness is an index that measures diversity along with species richness. The estimated Pielou’s evenness for the control (*p* = 0.6547) and treatment (*p* = 0.7494) samples indicated that there are no significant differences in the species evenness, which meant the species are distributed evenly within the control (Cpre vs. Cpost) and treatment (Tpre vs. Tpost) groups (Figure 1A,B). The Simpson index was used to calculate the relative abundance of the different species. The estimated Simpson index for the control (*p* = 0.8480) and treatment (*p* = 0.6547) groups were not significantly different, indicating that species richness was not significantly different among the groups (Figure 1C,D). However, the evenness and species richness were higher in the treatment than in the control group. The diversity richness of the oral microbiome was estimated using the Shannon index. The Kruskal–Wallis (pairwise) test identified the differences in the control (*p* = 0.5653) and treatment (*p* = 0.1797) groups. The results showed that even though the statistical differences were not observed in the control and treatment groups, there could be a difference in diversity for the treatment group compared to the control group (Figure 1E,F).

#### 3.2.2. Beta-Diversity

**Figure 2:**
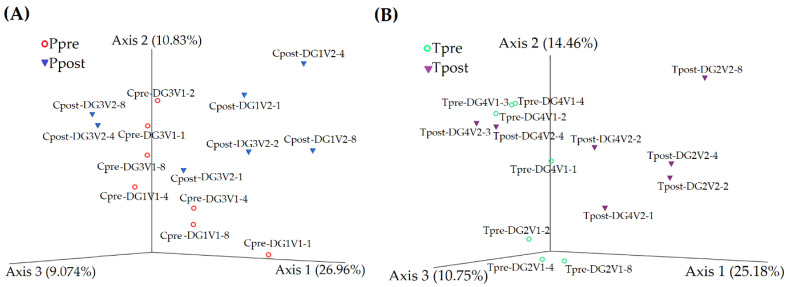
The inter-group relationships between the pre- and post-samples of control (Cpre vs. Cpost) (**A**) and treatment (Tpre vs. Tpost) (**B**) were calculated using the PCoA plot.

The similarity and variations in the microbiome were performed using PCoA, and unweighted UniFrac distances between the groups were represented. Cpre and Cpost samples were not completely separated from each other in 3D space. PCoA plot axis 1, 2, and 3 explained the variances in the microbial abundances in the samples as 26.96%, 10.83%, and 9.074%. However, the microbial load of the Cpost samples Cpost-DG3V2-1, Cpost-DG3V2-4, and Cpost-DG3V2-8 were located near the Cpre samples. They formed a cluster, which indicated that there could be a microbial similarity between the Cpre and Cpost samples, but the samples Cpost-DG1V2-1, Cpost-DG1V2-2, Cpost-DG1V2-4, and Cpost-DG1V2-8 were completely separated in the 3D space, which described that there could be a possible microbial difference in the Cpre and Cpost samples (Figure 2A).

Alternatively, the Tpre and Tpost samples were completely separated in 3D space except in Tpost-DG4V2-3 and Tpost-DG4V2-4, which were positioned near the Tpre samples (Tpre-DG4V1-2, Tpre-DG4V1-3, and Tpre-DG4V1-4). The results revealed that microbial similarity existed between the Tpre and Tpost samples. Whereas Tpost samples such as Tpost-DG2V2-2, Tpost-DG2V2-8, Tpost-DG4V2-2, and Tpost-DG4V2-4 are completely separated from the Tpre samples described that the increased microbial dissimilarity may exist between the Tpre and Tpost samples in the treatment group. PCoA plot axis 1, axis 2, and axis 3 explained the variances in the microbial abundances in the samples as 25.18%, 14.46%, and 10.75% (Figure 2B).

#### 3.2.3. Taxonomical Assignment

**Figure 3:**
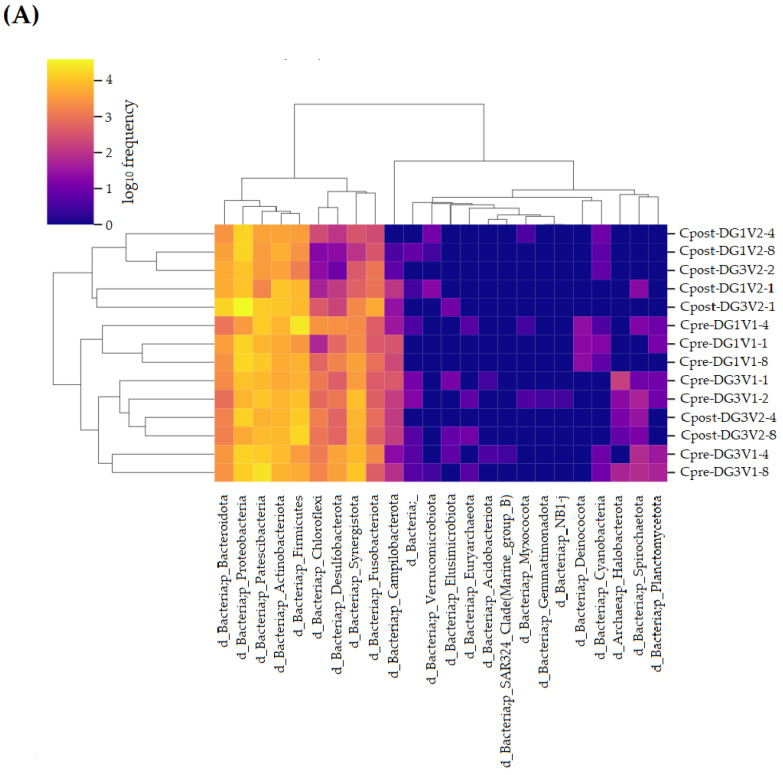

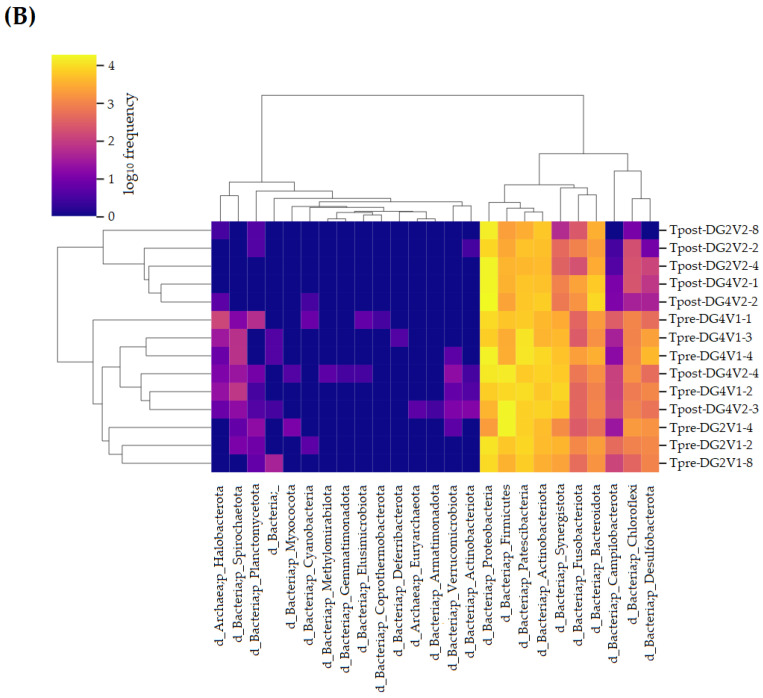
Heat map for the taxonomical assignment of experimental dog’s oral microbiome: (**A**) the taxonomical comparison of Cpre and Cpost samples of the control group; (**B**) the taxonomical comparison of Tpre and Tpost samples of the treatment group. Cpre and Tpre: pretreatment samples; Cpost and Tpost: post-treatment samples.

The taxonomy (from phylum to species) was estimated and assigned for the control and treatment samples and visualized as the heatmap (Figure 3).

##### Phylum

**Figure 4:**
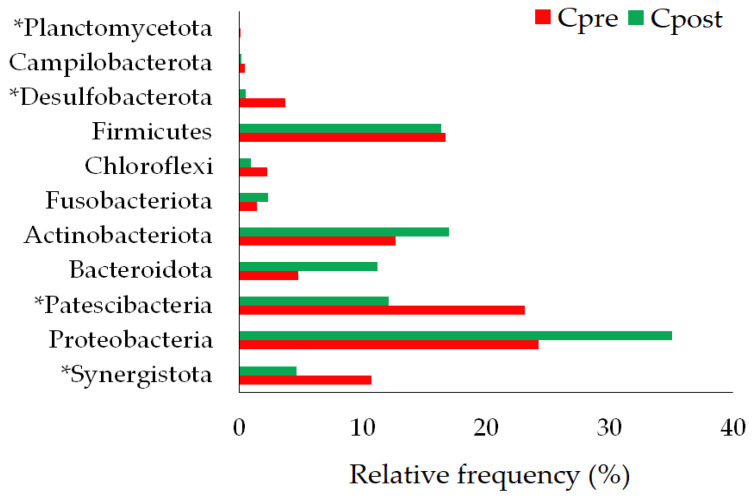
The phylum-level changes in the pre- and post-samples of control. * statistically significant (*p* ≤ 0.05).

**Figure 5:**
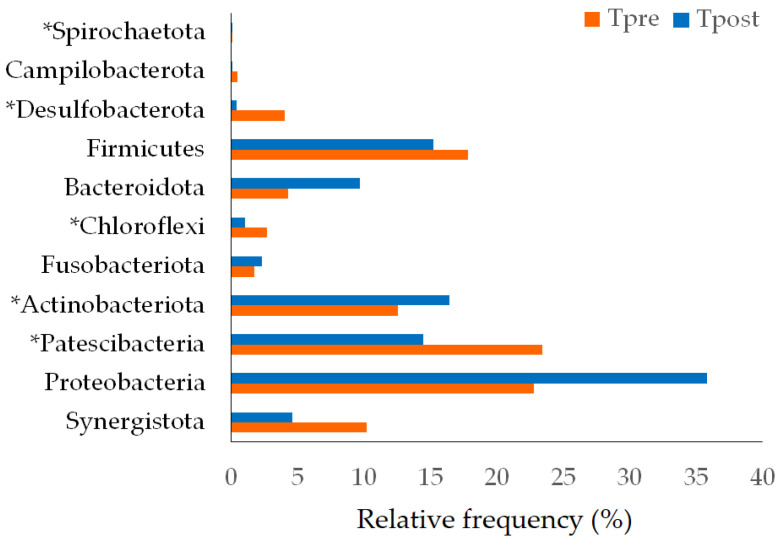
The phylum-level changes in the pre- and post-samples of the treatment group. * Statistically significant (*p* ≤ 0.05).

Phylum Synergistota, Proteobacteria, Patescibacteria, Bacteroidota, Actinobacteriota, Fusobacteriota, Chloroflexi, Firmicutes, Desulfobacterota, and Campilobacterota were detected in control (both in Cpre and Cpost) samples. The phylum Planctomycetota was detected in Cpre samples, whereas after four weeks, Planctomycetota was completely rooted out (Figure 4). The phylum Synergistota, Proteobacteria, Patescibacteria, Actinobacteriota, Fusobacteriota, Chloroflexi, Bacteroidota, Firmicutes, Desulfobacterota, Campilobacterota and Spirochaetota were detected in the treatment group (Figure 5).

The significant differences between the pre-and post-samples of control and treatment groups were calculated using the Wilcoxon signed-rank test and *t*-test (*p* ≤ 0.05 considered significant). The statistical analysis showed significant differences in Synergistota (*p* = 0.018), Patescibacteria (*p* = 0.000), Desulfobacterota (*p* = 0.0034), and Planctomycetota (*p* = 0.022) between the pre- and post-samples of the control group (Table 3). Similarly, Patescibacteria (*p* = 0.004), Actinobacteriota (*p* = 0.043), Chloroflexi (*p* = 0.0425), Desulfobacterota (*p* = 0.0014), and Spirochaetota (*p* = 0.022) showed the significant differences in the Tpost samples compared to Tpre samples. However, there were no significant microbial changes in phylum levels between the control and treatment groups (Table S5).

##### Genera

**Figure 6:**
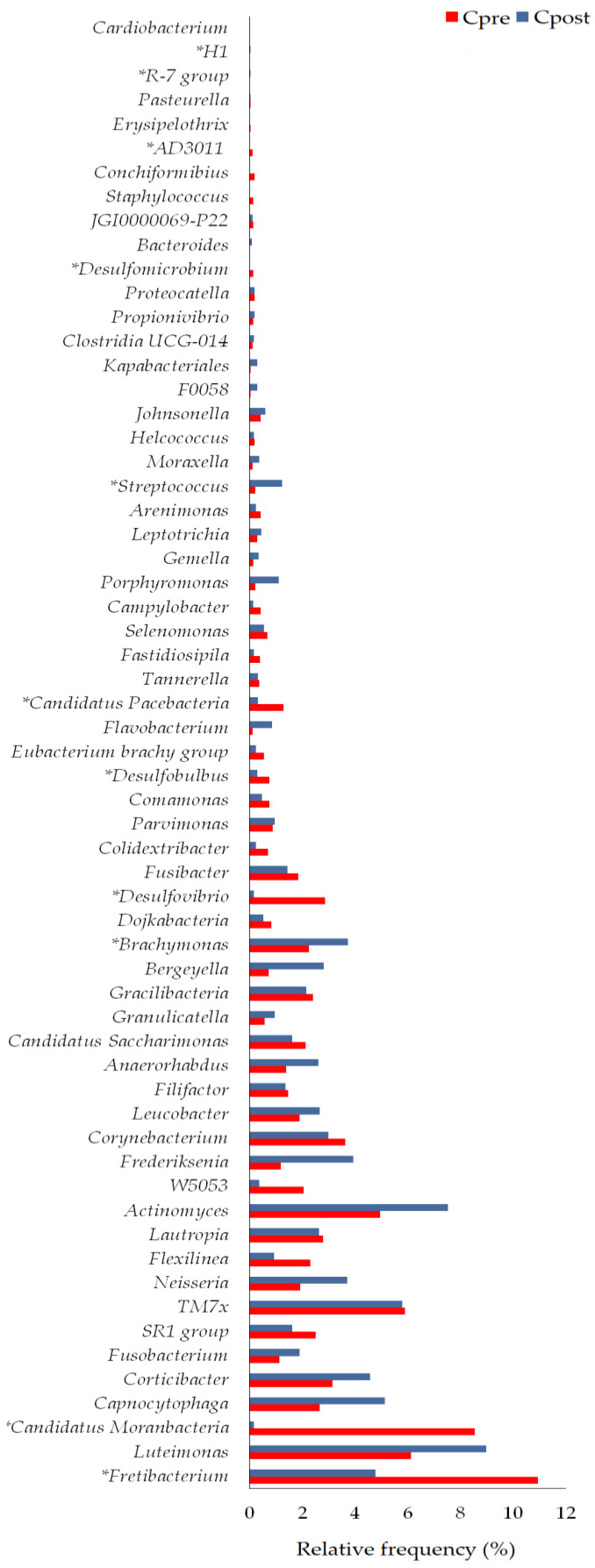
The genus level changes in the pre- and post-samples of control. * Statistically significant (*p* ≤ 0.05).

Sixty-one bacterial genera were detected in the control group (Figure 6); of them, the genus *Fretibacterium* (*p* = 0.018), *Candidatus Moranbacteria* (*p* = 0.028), *Brachymonas* (*p* = 0.0076), *Desulfovibrio* (*p* = 0.0091), *Desulfobulbus* (*p* = 0.0037), *Candidatus Pacebacteria* (*p* = 0.028), *Streptococcus* (*p* = 0.028), *Desulfomicrobium* (*p* = 0.0425), *AD3011* (*p* = 0.028), *R-7 group* (*p* = 0.018), and *H1* (*p* = 0.043) showed the significant differences in the Cpost samples compared to the Cpre samples (Table 3).

**Figure 7:**
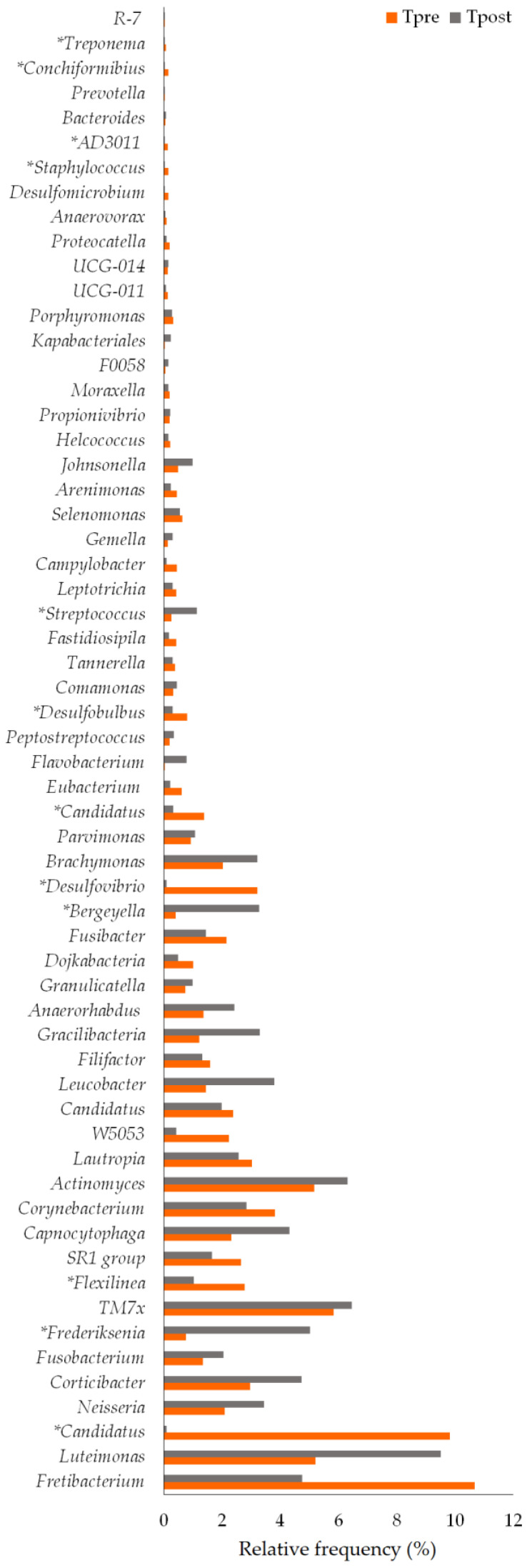
The genus level changes in the pre- and post-samples of the treatment group. * Statistically significant (*p* ≤ 0.05).

Sixty bacterial genera were detected in the treatment group (Figure 7). The genus such as *Candidatus* (*p* = 0.018), *Frederiksenia* (*p* = 0.028), *Flexilinea* (*p* = 0.043), *Bergeyella* (*p* = 0.043), *Desulfovibrio* (*p* = 0.003), *Desulfobulbus* (*p* = 0.01), *Streptococcus* (*p* = 0.028), *Staphylococcus* (*p* = 0.018), *AD3011* (*p* = 0.018), *Conchiformibius* (*p* = 0.043), and *Treponema* (*p* = 0.022) showed the significant differences in the Tpost samples compared to the Tpre samples (Table S5).

##### Species

**Figure 8:**
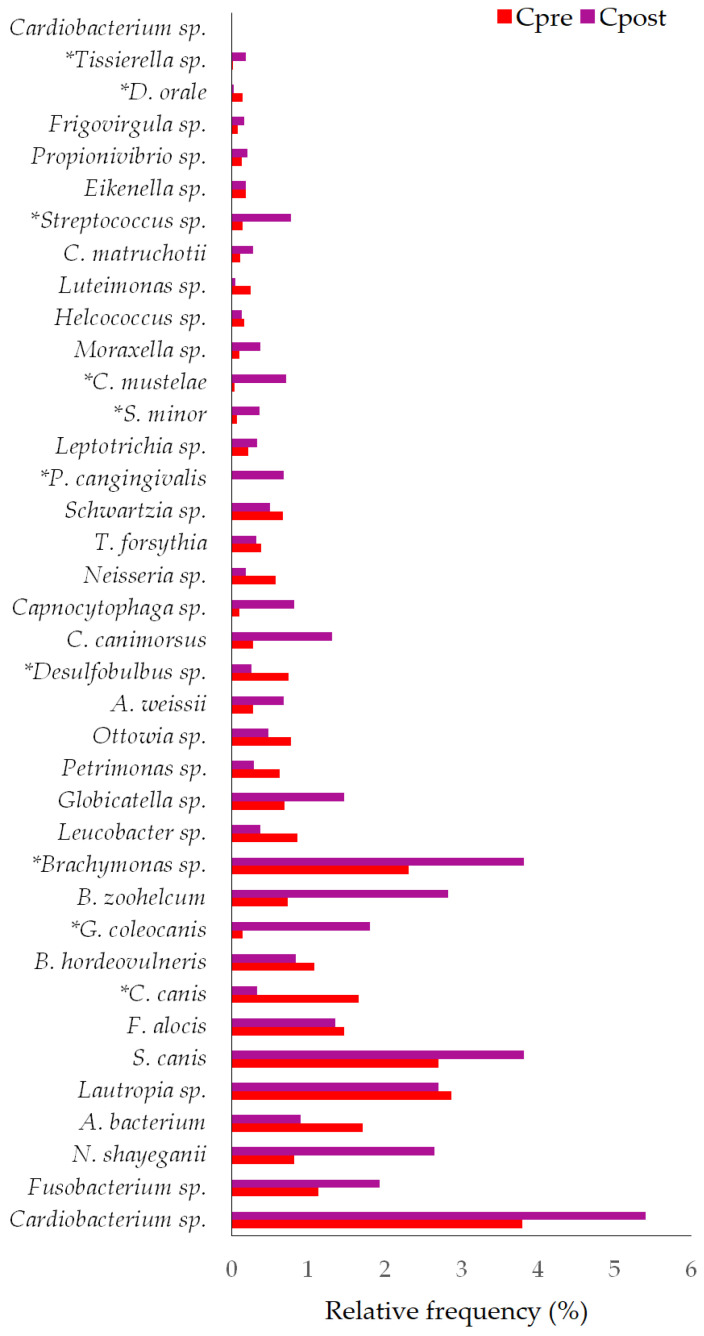
The species-level changes in the pre- and post-samples of control. * Statistically significant (*p* ≤ 0.05).

**Figure 9:**
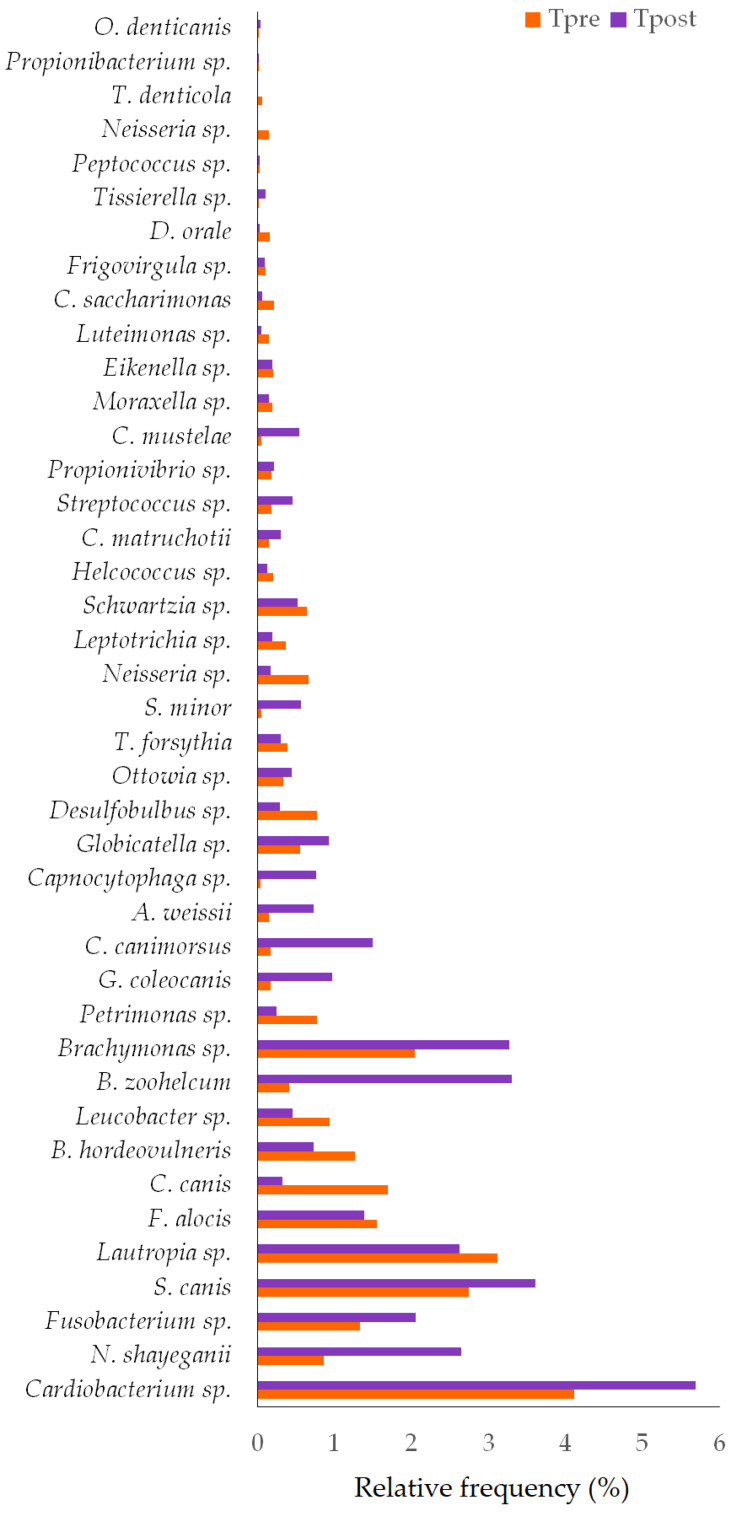
The species-level changes in the pre- and post-samples of the treatment group. * Statistically significant (*p* ≤ 0.05).

Thirty-eight and forty-one species were detected in the control (Figure 8) and treatment groups, respectively (Figure 9). The species such as *Corynebacterium canis* (*p* = 0.0180), *Gleimia coleocanis* (*p* = 0.0280), *Brachymonas* sp. (*p* = 0.0071), *Desulfobulbus* sp. (*p* = 0.0051), *Porphyromonas cangingivalis* (*p* = 0.022), *Streptococcus minor* (*p* = 0.0425), *Corynebacterium mustelae* (*p* = 0.028), *Streptococcus* sp. (*p* = 0.0425), *Desulfomicrobium orale* (*p* = 0.0425), and *Tissierella* sp. (*p* = 0.0280) were showed the statistical significance between the Cpre and Cpost samples (Table 3). Similarly, the species such as *Corynebacterium canis* (*p* = 0.018), *Buchananella hordeovulneris* (*p* = 0.018), *Bergeyella zoohelcum* (*p* = 0.0425), *Petrimonas* sp. (*p* = 0.018), *Desulfobulbus* sp. (*p* = 0.018), *Streptococcus minor* (*p* = 0.028), *Corynebacterium mustelae* (*p* = 0.028), *Neisseria* sp. (*p* = 0.0425), and *Treponema denticola* (*p* = 0.022) showed the significant differences in the post sample compared to its baseline values in the treatment group (Table S5).

## 4. Discussion

### 4.1. Clinical Parameters

At the baseline, the sex and weight of the dogs were not significantly different in both the control and treatment groups (Table 1). The effect of GAMS on GI, PI, and CI values was presented (Table 2). In the control group, no significant changes were observed in GI, and PI values, after six weeks of study. CI values were improved at seven positions compared to the control baseline. Whereas GI, PI, and CI values were improved at seven, one, and sixteen positions, compared to the baseline (pre) values in the treatment group (Table 2). The effect of mouth spray on PI value was not enhanced significantly at several positions. The results indicated that the GAMS usage improved the GI and CI scores effectively.

It is necessary to develop and improve the therapeutics to control and manage oral diseases, especially PD, in dogs [38]. Our GAMS showed better results in reducing the GI and CI, but a more prevalent impact of the PI score was not noted. Therefore, further tuning is required in the formulation to control the plaques.

### 4.2. The Changes in the Oral Microbiome

The sequencing study showed that the sequence’s reads were increased in the post-samples of control (Cpost) and treatment (Tpost) groups. Comparatively, the number of reads was less in Tpost samples than in Cpost samples, indicating that the oral bacterial abundances were decreased after GAMS treatment (Table S4).

The alpha diversity, such as Pielou’s evenness, Simpson, and Shannon index, stated that species richness, evenness, and diversity did not significantly differ (*p* ≤ 0.05) within the control (Figure 1A,C,E) and treatment (Figure 1B,D,F) groups. The beta diversity of the microbial community was represented as the PCoA plot, which stated that the microbial load of the treatment group’s samples was notably more varied than the control samples. The data indicated that the GAMS influences the oral microbial load in the experimental dogs (Figure 2A,B).

The changes in the oral microbiota of pre- and post-samples of the control and treatment groups were compared (Figure 4, Figure 5, Figure 6, Figure 7, Figure 8 and Figure 9). The results indicated significant changes within the group comparisons (Cpre vs. Cpost; Tpre vs. Tpost) (Table 3). No significant changes were observed between the control and treatment groups (Table S5).

#### 4.2.1. Changes in the Bacterial Phylum

Synergistota members were not majorly detected in the healthy oral samples, but the members of phylum Synergistota have a role in periodontitis [39]. The changes in the abundances of Patescibacteria and Desulfobacterota were observed in association with inflammatory bowel diseases and Crohn’s disease [40,41]. The 16S rDNA profiling stated that Actinobacteria was the oral inhabitant in healthy human and canine oral samples [42,43,44]. Dewhirst et al. stated that Chloroflexi was the dominant phylum in the canine subgingival plaques [45]. Spirochetes are related to periodontitis and could destroy gingival tissue [46]. Our study showed that the abundance of the above-mentioned phylum was altered in the control and treatment groups after the study period. The changes were significant within the group but insignificant between the groups (Figure 4 and Figure 5; Table 3; Table S5), indicating that GAMS was not shown any significant impact on the oral microbiota of dogs.

#### 4.2.2. Changes in Bacterial Genera

Significant changes were observed in the relative frequency of Fretibacterium, Candidatus Moranbacteria, Brachymonas, Desulfovibrio, Desulfobulbus, Candidatus Pacebacteria, Streptococcus, Desulfomicrobium, AD3011, R-7 group, and H1 between the Cpre and Cpost samples (Figure 6 and Table 3). Similarly, the relative frequency of Candidatus Moranbacteria, Frederiksenia, Flexilinea, Bergeyella, Desulfovibrio, Candidatus Pacebacteria, Desulfobulbus, Streptococcus, Staphylococcus, AD3011, Conchiformibius, and Treponema was varied significantly between the Tpre and Tpost samples (Figure 7 and Table 3).

*Bergeyella* is abundantly present in healthy felines [47]. The members of the genus *Staphylococcus* may transfer from animals to humans, and they are associated with several diseases and are found in the oral cavities of small animals [48,49]. The relative abundance of *Desulfomicrobium* was higher in the subgingival plaque of the healthy adult Beagle dogs [50]. *Frederiksenia* was commonly found in healthy dogs, and its relative abundance was increased after probiotic treatment [51]. Nises et al. reported that several phylotypes of oral *Treponema* species are common in dogs despite periodontal disease status [52]. In the present study, the relative frequency of the *Bergeyella* (*p* = 0.0425) and *Staphylococcus* (*p* = 0.018) was increased and decreased significantly in the Tpost samples, respectively. The relative abundance of *Frederiksenia* (*p* = 0.028) was increased significantly in the Tpost samples. *Treponema* was detected only in the treatment group (Figure 6 and Figure 7; Table 3 and Table S5).

*Desulfovibrio* genus was more abundantly present in the PD-affected dogs than in the healthy subjects [7]. *Desulfobulbus* genus is associated with a subgingival plaque of periodontitis [53]. The relative frequency of *Desulfovibrio* (*p* = 0.003) *and Desulfobulbus* (*p* = 0.01) was reduced significantly after GAMS usage (Figure 7).

*Fretibacterium* may involve periodontal pathogenesis in humans. The abundance of *Fretibacterium* was significantly higher in the periodontitis subjects than in the healthy humans [54]. In our study, the relative frequency of *Fretibacterium* was reduced in the Cpost (*p* = 0.018) and Tpost (*p* = 0.091) samples (Figure 6 and Figure 7), indicating that the reduction in *Fretibacterium* was not associated with the GAMS usage.

*Flexilinea* was abundantly present in the periodontitis sites of the periodontal patients [55]. The relative frequency of the *Flexilinea* was greatly reduced in the Tpost (*p* = 0.0425) samples (Figure 7), suggesting that GAMS usage may act against *Flexilinea*.

The results showed that GAMS could improve the oral microbiome by enhancing the normal oral flora. However, positive changes in some of the genera were observed in both control and treatment groups, so additional studies are required to demonstrate the protective effect of GAMS against PD.

#### 4.2.3. Changes in Bacterial Species

The species include *C. canis*, *G. coleocanis*, *Brachymonas* sp., *Desulfobulbus* sp., *P. cangingivalis*, *S. minor*, *C. mustelae*, *Streptococcus* sp., *D. orale*, and *Tissierella* sp. showed the significant changes in the control group compared to the baseline (Figure 8). *C. canis*, *B. hordeovulneris*, *B. zoohelcum*, *Petrimonas* sp., *Desulfobulbus* sp., *S. minor*, *C. mustelae*, *Neisseria* sp., and *T. denticola* showed significant changes in the treatment group compared to the baseline (Figure 9).

*C. canis* is more prevalent in dogs with periodontitis than in healthy dogs [56,57]. *C. canis* was also isolated from a patient’s wound caused by a dog bite [58]. *Desulfobulbus* sp. also causes PD [59]. *Neisseria* sp. is a commensal of the canine and feline oral cavities. However, *Neisseria* sp. is a potential cause of cutaneous infections in people secondary to animal bites [60]. The relative frequency of *C. canis* and *Desulfobulbus* sp. was reduced in both the control and treatment groups at the end of the study (Figure 8 and Figure 9). *Neisseria* sp. abundance was reduced significantly (*p* = 0.043) in the treatment group (Table 3).

The mouth spray containing gallic acid efficiently decreased the abundance of 22 species and increased the abundance of 17 species in cats [32]. The changes in some species were unexplainable, and their role in oral health diseases must be elucidated, and some species were found either in control or treatment samples. *Brachymonas* sp. may be the commensal oral bacteria that may prevent dental caries. *Brachymonas* sp. relative frequency was increased in the control and treatment groups (Figure 8 and Figure 9). *P. cangingivalis* is the most prevalent canine oral bacterium [57], which was detected only in the control group, and its relative frequency was increased after six weeks (Figure 8). *D. orale* was isolated from the subgingival plaque of a patient with periodontitis, which plays a potential etiopathogenetic role in PD [61,62]. The abundance of *D. orale* and Nicoletella species was found to be decreased in the post-treated experimental group when compared to its baseline values indicating that the mouth spray containing gallic acid could reduce the pathogen load in cats [32]. In the present study, *D. orale* relative frequency was reduced in the control and treatment groups (Figure 8 and Figure 9). *B. zoohelcum* causes rare but severe human clinical diseases, mostly arising from animal bites. Older adults may contract *Bergeyella* infections following continuous contact with dogs or cats without being bitten [63]. *B. zoohelcum* abundance was increased in both control and treatment groups (Figure 8 and Figure 9).

Like genus variations, species-level variations in the control and treatment groups need further studies to prove the impact of GAMS on the oral health of healthy dogs.

## 5. Conclusions

The study has limitations, such as fewer experimental subjects, short study duration, and metagenomic samples. The GAMS act well against the GI and CI than the PI. The microbiome analysis revealed that the GAMS usage altered the oral microbiota. Specifically, the abundance of the commensal bacterial phylum Actinobacteria and Chloroflexi, genera *Frederiksenia*, and *Bergeyella* was improved after six weeks of GAMS usage. The GAMS usage reduced the pathogenic bacterial species, including *Neisseria* sp., *C. canis*, *Desulfobulbus* sp., and *C. mustelae*. Moreover, some pathogenic bacterial abundances were increased at the end of the study. All the microbial variations were observed within the group. The inter-group analysis revealed that the changes were unrelated to GAMS usage. Further studies need to be carried out using more experimental subjects to confirm the effectiveness of GAMS. More metagenomic data are required to evidence the GMAS impact on the oral microbiome of healthy dogs.

## Figures and Tables

**Table 1:** Demographic data of the experimental dogs.

Characters	Control	Treatment	*p*-Value
Male	9	6	0.508
Female	10	13	
Weight (Mean ± SD)	15.29 ± 7.99	12.76 ± 5.14	0.175

**Table 2:** Median, interquartile (IQ) values, and comparison (pre- vs. post-treatment) of the gingival index (GI), plaque index (PI), and calculus index for control and treatment groups.

Parameters	Tooth Code	Position	Median (IQ Range: Q3–Q1)					
			Control			Treatment		
			Pre	Post	Comparison * (*p*-Value)	Pre	Post	Comparison * (*p*-Value)
Gingivitis index	104	1	0 (1)	0 (2)	0.414	0 (1)	0 (0)	0.256
		2	0 (1)	0 (1)	0.217	0.5 (1)	0 (1)	0.053
		3	0 (1)	0 (4)	0.705	0.5 (1)	0 (0)	0.014
	108	1	3 (7)	4 (6)	0.131	2 (3)	1 (4)	0.014
		2	4 (3)	5 (2)	0.073	3 (7)	1.5 (4)	0.045
		3	4 (6)	4 (5)	0.243	4 (0)	0.5 (1)	0.397
	204	1	1 (1)	1 (2)	0.083	0 (1)	0 (1)	0.414
		2	1 (1)	1 (0)	0.290	1 (2)	0 (2)	0.689
		3	1 (1)	1 (1)	0.123	0 (0)	0 (0)	0.000
	208	1	4 (8)	3 (7)	0.179	1 (3)	1 (4)	0.315
		2	4 (4)	5 (2)	0.256	1 (8)	1 (7)	0.121
		3	4 (6)	4 (5)	0.288	2 (0)	0 (0)	0.005
	304	1	0 (0)	0 (0)	0.954	0 (0)	0 (0)	0.951
		2	0 (0)	0 (0)	0.093	0 (1)	0 (0)	0.348
		3	0 (1)	0 (0)	0.387	0 (0)	0 (0)	0.563
	309	1	0 (1)	0 (1)	0.654	1 (1)	0 (0)	0.219
		2	0 (1)	0 (1)	0.102	0 (0)	0 (0)	0.967
		3	0 (0)	0 (1)	0.317	0 (0)	0 (0)	0.317
	404	1	0 (0)	0 (0)	0.617	0 (0)	0 (0)	0.157
		2	0 (0)	0 (0)	0.479	0 (1)	0 (1)	0.317
		3	0 (1)	0 (0)	0.824	0 (1)	0 (1)	0.654
	409	1	0 (1)	0 (1)	0.705	1 (0)	0 (0)	0.615
		2	0 (1)	0 (1)	0.713	0 (0)	0 (0)	0.000
		3	0 (1)	0 (0)	0.291	0 (0)	0 (0)	0.000
Plaque index	104	1	4 (11)	3 (7)	0.118	4 (6)	4 (4)	0.584
		2	0 (3)	0 (1)	0.280	0 (2)	1 (2)	0.645
	108	1	4 (3)	4 (6)	0.755	2 (3)	3 (2)	0.107
		2	4 (3)	4 (7)	0.534	3.5 (2)	4 (3)	0.089
	204	1	6 (11)	4 (6)	0.128	8 (9)	5 (5)	0.029
		2	0 (1)	0 (1)	1.000	1 (3)	1.5 (3)	0.253
	208	1	4 (5)	4 (4)	0.180	2 (7)	4 (5)	0.162
		2	4 (4)	4 (3)	0.214	2 (7)	4 (5)	0.161
	304	1	2 (3)	1 (4)	0.085	2.5 (5)	3.5 (5)	0.965
		2	0 (1)	0 (1)	0.260	1 (2)	0 (2)	0.980
	309	1	3 (2)	2 (2)	0.058	2.5 (2)	2.5 (3)	0.508
		2	0 (2)	1 (2)	0.918	1 (1)	1 (1)	0.212
	404	1	2 (5)	2 (4)	0.304	2 (3)	2 (6)	0.562
		2	0 (0)	0 (0)	0.655	0 (0)	0 (0)	0.210
	409	1	2 (3)	2 (3)	0.596	2 (2)	2.5 (3)	0.321
		2	0 (1)	1 (1)	0.949	0 (1)	1 (1)	0.212
Calculus index	104	1	0 (1)	0 (2)	0.882	0 (1)	0 (0)	0.115
		2	0 (1)	0 (1)	0.605	0.5 (1)	0 (1)	0.066
		3	0 (1)	0 (4)	0.089	0.5 (1)	0 (0)	0.021
	108	1	3 (7)	4 (6)	0.028	2 (3)	1 (4)	0.013
		2	4 (3)	5 (2)	0.004	3 (7)	1.5 (4)	0.049
		3	4 (6)	4 (5)	0.015	4 (4)	0.5 (4)	0.025
	204	1	1 (1)	1 (2)	0.361	0 (1)	0 (1)	0.000
		2	1 (1)	1 (0)	0.401	1 (2)	0 (2)	0.000
		3	1 (1)	1 (1)	0.008	0 (1)	0 (0)	0.003
	208	1	4 (8)	3 (7)	0.034	1 (3)	1 (4)	0.014
		2	4 (4)	5 (2)	0.008	1 (8)	1 (7)	0.007
		3	4 (6)	4 (5)	0.027	2 (4)	0 (4)	0.028
	304	1	0 (0)	0 (0)	0.157	0 (0)	0 (0)	0.157
		2	0 (0)	0 (0)	0.093	0 (1)	0 (0)	0.045
		3	0 (1)	0 (0)	0.204	0 (0)	0 (0)	0.083
	309	1	0 (1)	0 (1)	0.980	1 (1)	0 (0)	0.032
		2	0 (1)	0 (1)	0.693	0 (0)	0 (0)	0.045
		3	0 (0)	0 (1)	0.317	0 (0)	0 (0)	0.157
	404	1	0 (0)	0 (0)	0.157	0 (0)	0 (0)	0.317
		2	0 (0)	0 (0)	0.025	0 (1)	0 (1)	0.083
		3	0 (1)	0 (0)	0.098	0 (1)	0 (0)	0.014
	409	1	0 (1)	0 (1)	0.164	1 (0)	0 (0)	0.031
		2	0 (1)	0 (1)	0.053	0 (0)	0 (0)	0.519
		3	0 (1)	0 (0)	0.317	0 (0)	0 (0)	0.000

* Wilcoxon signed-rank test. Significance level (α): *p* ≤ 0.05.

**Table 3:** The statistically significant difference in the phylum, genera, and species between pre- and post-samples of control and treatment groups.

Control (Pre- vs. Post-Samples)		Treatment (Pre- vs. Post-Samples)	
Taxonomy	*p*-Value *	Taxonomy	*p*-Value *
Phylum			
Synergistota	0.018	Spirochaetota	0.022
Patescibacteria	0.000	Patescibacteria	0.004
Desulfobacterota	0.003	Desulfobacterota	0.001
Planctomycetota	0.022	Actinobacteriota	0.043
		Chloroflexi	0.043
Genera			
*Fretibacterium*	0.018	*Candidatus*	0.018
*Candidatus moranbacteria*	0.028	*Frederiksenia*	0.028
*Brachymonas*	0.007	*Flexilinea*	0.043
*Desulfovibrio*	0.009	*Desulfovibrio*	0.003
*Desulfobulbus*	0.004	*Desulfobulbus*	0.010
*Candidatus Pacebacteria*	0.028	*Bergeyella*	0.043
*Streptococcus*	0.028	*Streptococcus*	0.028
*Desulfomicrobium*	0.043	*Candidatus*	0.018
*AD3011*	0.028	*AD3011*	0.018
*R7 group*	0.018	*Staphylococcus*	0.018
*H1*	0.043	*Conchiformibius*	0.043
		*Treponema*	0.022
Species			
*C. canis*	0.018	*C. canis*	0.018
*G. coleocanis*	0.028	*B. hordeovulneris*	0.018
*Brachymonas* sp.	0.007	*B. zoohelcum*	0.043
*Desulfobulbus* sp.	0.005	*Desulfobulbus* sp.	0.018
*P. cangingivalis*	0.022	*Petrimonas* sp.	0.018
*S. minor*	0.042	*S. minor*	0.028
*C. mustelae*	0.028	*C. mustelae*	0.028
*Streptococcus* sp.	0.042	*Neisseria* sp.	0.043
*D. orale*	0.042	*T. denticola*	0.022
*Tissierella* sp.	0.028		

* *p* ≤ 0.05 was considered significant.

## Data Availability

All the related data have been provided in the manuscript.

## Supplementary Materials

The following supporting information can be downloaded at: https://www.mdpi.com/article/10.3390/vetsci10070424/s1, Table S1: Logan and Boyce index for determining the plaque index; Table S2: Warrick and Gorrel method to determine the calculus index; Table S3: Löe and Silness index to determine the gingivitis index; Table S4: The estimated sequences in the control and treatment samples; Table S5: The average and SD of the relative frequency of taxonomical parameters and their comparison between the groups.
