# Supplementary material for: Influence of Gallic Acid-Containing Mouth Spray on Dental Health and Oral Microbiota of Healthy Dogs: A Pilot Study
Source: Vet Sci. 2023 Jun 30;10(7):424. doi: 10.3390/vetsci10070424 (PMC10385331; doi:10.3390/vetsci10070424)
Supplement: Supplementary file 1 [file vetsci-10-00424-s001.zip › vetsci-2367735-supplementary.pdf]

Table S1. Logan and Boyce index for determining the plaque index.

Coverage scores		Thickness scores	
0	None observed	0	None observed
1	< 25%	1 (light)	Pink to light red
2	25 to 49%	2 (medium)	Red
3	50 to 75%	3 (heavy)	Dark red
4	>75%		

Table S2. Warrick and Gorrel method to determine the calculus index.

Coverage scores		Thickness scores	
0	None observed	0	None observed
1	< 25%	1	< 0.5 mm
2	25 to 49%	2	0.5 to 1 mm
3	50 to 75%	3	>1
4	>75%		

Table S3. Löe and Silness index to determine the gingivitis index.

Score	Criteria
0	No inflammation
1	Mild inflammation; Slight color change; Slight edema; No bleeding on probing.
2	Moderate inflammation; Redness; Bleeding on probing
3	Severe inflammation; Marked redness; Ulceration; Spontaneous bleeding

Table S4. The estimated sequences in the control and treatment samples.

Samples-id	Input	Filtered	Passed filter (%)	Denoised	Merged	Input merged (%)	non-chimeric	Non-chimeric (%)
Control								
Cpre-DG1V1-1	48812	40519	83.01	39905	38532	78.94	37830	77.50
Cpre-DG1V1-4	75090	59931	79.81	59016	57584	76.69	56078	74.68
Cpre-DG1V1-8	67602	54111	80.04	53439	51499	76.18	49814	73.69
Cpre-DG3V1-1	48493	41600	85.79	40616	38429	79.25	36562	75.40
Cpre-DG3V1-2	59026	48734	82.56	48174	47039	79.69	45580	77.22
Cpre-DG3V1-4	73460	61594	83.85	60798	58954	80.25	57028	77.63
Cpre-DG3V1-8	82927	71921	86.73	71131	69636	83.97	67538	81.44
Cpost-DG3V2-1	132133	107082	81.04	105846	101944	77.15	93886	71.05
Cpost-DG3V2-2	35683	29194	81.81	28670	27282	76.46	26255	73.58
Cpost-DG3V2-4	74497	62955	84.51	61215	57183	76.76	51474	69.10
Cpost-DG3V2-8	66150	55275	83.56	54170	51883	78.43	47933	72.46
Cpost-DG1V2-1	51668	40954	79.26	40405	39165	75.80	38250	74.03
Cpost-DG1V2-4	39927	32477	81.34	32186	31366	78.56	30966	77.56
Cpost-DG1V2-8	42217	34148	80.89	33803	32951	78.05	32440	76.84
Treatment								
Tpre-DG2V1-2	52520	42389	80.71	41663	40329	76.79	39054	74.36
Tpre-DG2V1-4	45590	37156	81.5	36586	35705	78.32	35010	76.79
Tpre-DG2V1-8	36860	30470	82.66	30045	29009	78.70	28036	76.06
Tpre-DG4V1-1	41598	35358	85	34631	32950	79.21	31792	76.43
Tpre-DG4V1-2	55474	45746	82.46	45030	43457	78.34	42254	76.17
Tpre-DG4V1-3	42917	36405	84.83	35928	34870	81.25	34098	79.45
Tpre-DG4V1-4	68965	57676	83.63	57057	55571	80.58	54615	79.19
Tpost-DG4V2-1	53527	44781	83.66	44113	42366	79.15	40355	75.39
Tpost-DG4V2-2	56669	47288	83.45	46719	45057	79.51	44029	77.70
Tpost-DG4V2-3	61098	48543	79.45	47621	45842	75.03	42864	70.16
Tpost-DG4V2-4	63373	52860	83.41	52113	50708	80.02	49699	78.42
Tpost-DG2V2-2	33941	26110	76.93	25697	24898	73.36	24504	72.20
Tpost-DG2V2-4	43687	36046	82.51	35428	33664	77.06	31619	72.38
Tpost-DG2V2-8	39422	32600	82.69	32133	30691	77.85	28721	72.86

Table S5. The average and SD of the relative frequency of taxonomical parameters and their comparison between the groups.

Taxonomy	Cpre Vs. Tpre (Mean ± SD)		Cpost Vs. Tpost (Mean ± SD)		Differences (Mean ± SD)		*p*-value
	**Cpre**	**Tpre**	**Cpost**	**Tpost**	**Control**	**Treatment**	
Phylum							
Synergistota	10.70 ± 5.71	10.22 ± 4.69	4.59 ± 5.77	4.60 ± 5.59	-6.11 ± 5.84	-5.61 ± 6.71	0.848
Proteobacteria	24.27 ± 11.94	22.75 ± 9.48	35.03 ± 14.24	35.83 ± 14.85	10.76 ± 20.37	13.07 ± 18.77	0.749
Patescibacteria	23.12 ± 4.98	23.44 ± 4.47	12.09 ± 3.94	14.43 ± 3.75	-11.03 ± 3.18	-9.00 ± 5.31	0.482
Bacteroidota	4.78 ± 2.82	4.30 ± 1.80	11.18 ± 6.55	9.67 ± 6.22	6.40 ± 6.89	5.37 ± 6.94	0.749
Actinobacteriota	12.64 ± 1.87	12.56 ± 1.83	16.97 ± 5.32	16.42 ± 2.92	4.33 ± 5.81	3.85 ± 4.26	0.949
Fusobacteriota	1.40 ± 0.92	1.70 ± 1.16	2.32 ± 1.80	2.32 ± 1.89	0.93 ± 2.14	0.61 ± 2.23	0.794
Chloroflexi	2.25 ± 1.53	2.66 ± 1.25	0.90 ± 1.10	1.01 ± 1.09	-1.34 ± 1.65	-1.65 ± 1.65	0.730
Firmicutes	16.70 ± 13.43	17.81 ± 13.19	16.32 ± 10.88	15.21 ± 11.76	-0.38 ± 21.06	-2.60 ± 21.12	0.949
Desulfobacterota	3.70 ± 2.23	4.04 ± 2.13	0.47 ± 0.49	0.42 ± 0.51	-3.24 ± 1.83	-3.62 ± 1.71	0.655
Campilobacterota	0.42 ± 0.41	0.43 ± 0.45	0.13 ± 0.14	0.08 ± 0.13	-0.29 ± 0.44	-0.35 ± 0.53	0.848
Genera							
Fretibacterium	10.94 ± 5.72	10.67 ± 4.90	4.76 ± 6.04	4.75 ± 5.82	-6.17 ± 6.05	-5.92 ± 7.17	0.848
Luteimonas	6.12 ± 4.16	5.20 ± 3.78	8.97 ± 7.03	9.51 ± 7.73	2.84 ± 6.87	4.31 ± 6.99	0.848
Candidatus	11.94 ± 7.75	13.58 ± 5.74	2.08 ± 1.88	2.39 ± 1.78	-9.86 ± 6.37	-11.18 ± 4.60	0.565
Capnocytophaga	2.66 ±1.97	2.31 ± 1.38	5.13 ± 4.97	4.32 ± 4.75	2.47 ± 5.07	2.01 ± 4.91	0.749
Corticibacter	3.15 ± 2.21	2.96 ± 2.15	4.57 ± 3.18	4.72 ± 3.21	1.42 ± 4.00	1.76 ± 3.74	0.749
Fusobacterium	1.14 ± 0.77	1.34 ± 0.93	1.90 ± 1.83	2.04 ± 1.96	0.76 ± 2.12	0.70 ± 2.23	0.848
TM7x	5.88 ±1.37	5.83 ± 1.31	5.78 ± 3.42	6.45 ± 2.98	-0.10 ± 3.78	0.62 ± 3.11	0.701
Neisseria	1.93 ± 1.89	2.08 ± 2.01	3.70 ± 1.83	3.43 ± 1.99	1.77 ± 2.07	1.35 ± 2.46	0.406
Flexilinea	2.31 ± 1.59	2.77 ± 1.32	0.93 ± 1.14	1.03 ± 1.13	-1.37 ± 1.74	-1.73 ± 1.76	0.482
Lautropia	2.78 ±2.58	3.03 ± 2.95	2.63 ± 2.82	2.57 ± 2.61	-0.14 ± 4.71	-0.46 ± 4.88	0.749
Actinomyces	4.94 ± 1.15	5.17 ± 1.36	7.52 ± 4.71	6.31 ± 3.13	2.58 ± 5.28	1.14 ± 3.87	0.749
W5053	2.05 ± 3.31	2.22 ± 3.41	0.37 ± 0.73	0.41 ± 0.81	-1.68 ± 3.58	-1.80 ± 3.73	0.848
Frederiksenia	1.19 ± 1.41	0.74 ± 0.94	3.92 ± 4.93	5.01 ± 5.01	2.73 ± 4.96	4.26 ± 4.66	0.482
Corynebacterium	3.63 ± 1.07	3.81 ± 0.97	2.99 ± 1.90	2.83 ± 2.0	-0.64 ± 1.48	-0.97 ± 1.60	0.482
Leucobacter	1.88 ± 1.09	1.45 ± 0.72	2.65 ± 1.96	3.79 ± 3.48	0.76 ± 2.45	2.34 ± 3.92	0.406
Filifactor	1.45 ± 2.92	1.58 ± 3.03	1.35 ± 2.37	1.33 ± 2.43	-0.10 ± 4.30	-0.25 ± 4.45	0.947
Anaerorhabdus	1.38 ± 0.59	1.37 ± 0.63	2.60 ± 1.60	2.41 ± 1.26	1.22 ± 1.68	1.04 ± 1.24	0.848

Granulicatella	0.58 ± 0.57	0.74 ± 0.59	0.95 ± 1.04	0.97 ± 0.96	0.37 ± 1.42	0.23 ± 1.47	0.949
Gracilibacteria	2.40 ± 3.15	1.20 ± 0.79	2.14 ± 2.49	3.29 ± 3.73	-0.26 ± 4.17	2.09 ± 3.78	0.482
Bergeyella	0.72 ± 0.99	0.40 ± 0.65	2.81 ± 2.76	3.27 ± 2.54	2.08 ± 2.79	2.87 ± 2.38	0.565
Brachymonas	2.26 ± 1.13	2.01 ± 0.77	3.73 ± 1.37	3.21 ± 1.44	1.46 ± 0.98	1.19 ± 1.32	0.482
Dojkabacteria	0.82 ± 0.79	1.01 ± 0.79	0.52 ± 0.74	0.48 ± 0.71	-0.29 ± 0.81	-0.53 ± 0.73	0.576
Desulfovibrio	2.87 ± 2.00	3.21 ± 1.85	0.16 ± 0.21	0.09 ± 0.16	-2.70 ± 1.89	-3.11 ± 1.74	0.684
Fusibacter	1.85 ± 2.46	2.14 ± 2.59	1.44 ± 1.18	1.43 ± 1.2	-0.40 ± 2.97	-0.70 ± 3.16	0.861
Parvimonas	0.89 ± 1.44	0.93 ± 1.51	0.96 ± 1.58	1.07 ± 1.59	0.07 ± 2.47	0.14 ± 2.58	0.961
Comamonas	0.76 ± 1.06	0.33 ± 0.26	0.47 ± 0.40	0.44 ± 0.46	-0.28 ± 1.13	0.10 ± 0.59	0.565
Desulfobulbus	0.74 ± 0.52	0.79 ± 0.60	0.28 ± 0.41	0.31 ± 0.43	-0.46 ± 0.26	-0.48 ± 0.34	0.893
Eubacterium	0.54 ± 0.80	0.62 ± 0.82	0.23 ± 0.40	0.21 ± 0.39	-0.31 ± 0.98	-0.41 ± 1.00	0.565
Flavobacterium	0.10 ± 0.15	0.04 ± 0.05	0.84 ± 1.53	0.77 ± 1.42	0.74 ± 1.55	0.73 ± 1.42	0.848
Tannerella	0.37 ± 0.26	0.37 ± 0.21	0.31 ± 0.19	0.29 ± 0.15	-0.06 ± 0.34	-0.08 ± 0.27	0.655
Fastidiosipila	0.40 ± 0.29	0.42 ± 0.26	0.15 ± 0.25	0.16 ± 0.27	-0.24 ± 0.44	-0.26 ± 0.44	0.942
Selenomonas	0.68 ± 0.74	0.64 ± 0.78	0.55 ± 1.01	0.56 ± 0.98	-0.12 ± 0.52	-0.08 ± 0.50	0.871
Campylobacter	0.42 ± 0.44	0.44 ± 0.49	0.13 ± 0.14	0.08 ± 0.13	-0.29 ± 0.48	-0.35 ± 0.57	0.848
Porphyromonas	0.22 ± 0.37	0.32 ± 0.60	1.11 ± 2.02	0.28 ± 0.35	0.88 ± 1.66	-0.03 ± 0.80	0.277
Gemella	0.15 ± 0.34	0.14 ± 0.31	0.33 ± 0.60	0.30 ± 0.71	0.18 ± 0.76	0.16 ± 0.83	0.963
Leptotrichia	0.28 ± 0.25	0.42 ± 0.34	0.44 ± 0.26	0.30 ± 0.21	0.16 ± 0.39	-0.11 ± 0.43	0.277
Arenimonas	0.40 ± 0.48	0.43 ± 0.46	0.24 ± 0.41	0.24 ± 0.42	-0.16 ± 0.64	-0.19 ± 0.64	0.942
Streptococcus	0.22 ± 0.08	0.26 ± 0.09	1.24 ± 0.86	1.12 ± 0.65	1.01 ± 0.89	0.86 ± 0.68	0.848
Moraxella	0.10 ± 0.09	0.19 ± 0.21	0.37 ± 0.55	0.15 ± 0.14	0.27 ± 0.56	-0.04 ± 0.24	0.277
Helcococcus	0.18 ± 0.17	0.22 ± 0.15	0.16 ± 0.24	0.15 ± 0.21	-0.02 ± 0.28	-0.07 ± 0.29	0.741
Johnsonella	0.42 ± 0.30	0.49 ± 0.50	0.59 ± 0.75	0.99 ± 1.62	0.16 ± 0.96	0.49 ± 1.91	0.949
F0058	0.04 ± 0.06	0.05 ± 0.07	0.29 ± 0.43	0.16 ± 0.29	0.24 ± 0.45	0.10 ± 0.33	0.655
UCG-014	0.10 ± 0.09	0.13 ± 0.08	0.15 ± 0.19	0.15 ± 0.22	0.04 ± 0.25	0.01 ± 0.27	0.655
Propionivibrio	0.14 ± 0.09	0.18 ± 0.11	0.20 ± 0.12	0.21 ± 0.13	0.06 ± 0.09	0.02 ± 0.12	0.565
Proteocatella	0.18 ± 0.13	0.20 ± 0.16	0.18 ± 0.23	0.09 ± 0.06	-0.00 ± 0.28	-0.11 ± 0.16	0.384
Desulfomicrobium	0.14 ± 0.16	0.15 ± 0.19	0.02 ± 0.02	0.02 ± 0.02	-0.11 ± 0.15	-0.13 ± 0.18	0.949
Bacteroides	0.02 ± 0.02	0.05 ± 0.05	0.10 ± 0.11	0.08 ± 0.11	0.07 ± 0.09	0.02 ± 0.11	0.277
Staphylococcus	0.14 ± 0.33	0.14 ± 0.31	0.01 ± 0.03	0.00 ± 0.00	-0.13 ± 0.33	-0.14 ± 0.31	0.225
Conchiformibius	0.20 ± 0.21	0.14 ± 0.13	0.02 ± 0.03	0.02 ± 0.03	-0.17 ± 0.20	-0.12 ± 0.13	0.848
R-7	0.01 ± 0.00	0.03 ± 0.02	0.04 ± 0.03	0.03 ± 0.03	0.03 ± 0.03	0.00 ± 0.04	0.025

Species							
Cardiobacterium sp.	3.80 ± 1.94	4.11 ± 2.04	10.82 ± 5.03	5.68 ± 2.61	7.01 ± 5.28	1.57 ± 2.82	0.025
Fusobacterium sp.	1.13 ± 0.74	1.33 ± 0.91	1.93 ± 1.86	2.05 ± 1.99	0.80 ± 2.12	0.72 ± 2.23	0.848
N. shayeganii	0.81 ± 0.48	0.86 ± 0.45	2.65 ± 2.00	2.64 ± 1.98	1.83 ± 2.39	1.78 ± 2.31	0.848
Lautropia sp.	2.87 ± 2.69	3.11 ± 3.06	2.70 ± 2.92	2.62 ± 2.70	-0.17 ± 4.88	-0.48 ± 5.04	0.749
S. canis	2.70 ± 0.81	2.74 ± 0.98	3.82 ± 1.65	3.60 ± 1.00	1.12 ± 1.97	0.85 ± 1.88	0.802
Filifactor alocis	1.47 ± 3.12	1.55 ± 3.22	1.35 ± 2.55	1.38 ± 2.64	-0.11 ± 4.56	-0.16 ± 4.72	0.985
C. canis	1.66 ± 1.45	1.69 ± 1.25	0.33 ± 0.64	0.32 ± 0.67	-1.32 ± 1.45	-1.36 ± 1.20	0.565
B. hordeovulneris	1.08 ± 0.48	1.27 ± 0.32	0.83 ± 0.53	0.72 ± 0.37	-0.24 ± 0.68	-0.54 ± 0.24	0.482
G. coleocanis	0.14 ± 0.12	0.17 ± 0.15	1.80 ± 2.72	0.97 ± 1.99	1.65 ± 2.69	0.80 ± 1.89	0.655
B. zoohelcum	0.73 ± 0.99	0.41 ± 0.66	2.82 ± 2.80	3.30 ± 2.55	2.09 ± 2.81	2.89 ± 2.39	0.565
Brachymonas sp.	2.31 ± 1.16	2.04 ± 0.77	3.82 ± 1.41	3.27 ± 1.47	1.51 ± 0.99	1.23 ± 1.33	0.482
Leucobacter sp.	0.86 ± 0.72	0.94 ± 0.58	0.38 ± 0.63	0.46 ± 0.80	-0.48 ± 0.86	-0.47 ± 0.94	0.996
Globicatella sp.	0.69 ± 0.42	0.55 ± 0.20	1.46 ± 1.12	0.92 ± 0.66	0.77 ± 0.93	0.37 ± 0.74	0.338
Petrimonas sp.	0.62 ± 0.75	0.77 ± 0.72	0.28 ± 0.24	0.25 ± 0.25	-0.34 ± 0.57	-0.52 ± 0.52	0.277
Ottowia sp.	0.77 ± 1.08	0.33 ± 0.26	0.48 ± 0.41	0.44 ± 0.47	-0.29 ± 1.15	0.10 ± 0.60	0.431
A. weissii	0.27 ± 0.37	0.15 ± 0.2	0.68 ± 0.95	0.73 ± 0.90	0.40 ± 1.13	0.57 ± 0.95	0.759
Desulfobulbus sp.	0.74 ± 0.54	0.77 ± 0.61	0.26 ± 0.36	0.29 ± 0.38	-0.47 ± 0.29	-0.48 ± 0.36	0.949
C. canimorsus	0.27 ± 0.36	0.16 ± 0.21	1.31 ± 1.52	1.49 ± 1.46	1.03 ± 1.68	1.32 ± 1.53	0.949
Capnocytophaga sp.	0.10 ± 0.16	0.03 ± 0.05	0.82 ± 1.56	0.76 ± 1.44	0.72 ± 1.58	0.72 ± 1.44	0.655
Neisseria sp.	0.58 ± 1.00	0.66 ± 0.99	0.18 ± 0.25	0.17 ± 0.23	-0.39 ± 1.07	-0.49 ± 1.06	0.749
T. forsythia	0.39 ± 0.28	0.39 ± 0.2	0.32 ± 0.20	0.30 ± 0.16	-0.06 ± 0.37	-0.08 ± 0.29	0.655
Schwartzia sp.	0.66 ± 0.73	0.64 ± 0.78	0.50 ± 0.89	0.51 ± 0.87	-0.16 ± 0.43	-0.12 ± 0.49	0.868
Leptotrichia sp.	0.22 ± 0.24	0.36 ± 0.35	0.33 ± 0.31	0.19 ± 0.23	0.11 ± 0.46	-0.17 ± 0.47	0.338
S. minor	0.07 ± 0.07	0.05 ± 0.07	0.36 ± 0.38	0.56 ± 0.58	0.29 ± 0.40	0.50 ± 0.58	0.406
C. mustelae	0.03 ± 0.04	0.05 ± 0.06	0.71 ± 0.72	0.54 ± 0.45	0.67 ± 0.73	0.49 ± 0.46	0.749
Moraxella sp.	0.10 ± 0.09	0.18 ± 0.22	0.38 ± 0.57	0.15 ± 0.15	0.27 ± 0.57	-0.03 ± 0.25	0.229
Helcococcus sp.	0.16 ± 0.18	0.20 ± 0.18	0.13 ± 0.20	0.13 ± 0.18	-0.02 ± 0.27	-0.07 ± 0.29	0.761
Luteimonas sp.	0.25 ± 0.28	0.15 ± 0.12	0.05 ± 0.06	0.04 ± 0.05	-0.20 ± 0.32	-0.10 ± 0.17	0.749
C. matruchotii	0.11 ± 0.11	0.14 ± 0.11	0.27 ± 0.36	0.30 ± 0.39	0.16 ± 0.37	0.16 ± 0.37	1.000
Streptococcus sp.	0.14 ± 0.07	0.18 ± 0.05	0.77 ± 0.88	0.45 ± 0.44	0.63 ± 0.93	0.27 ± 0.46	0.565
Eikenella sp.	0.18 ± 0.19	0.21 ± 0.17	0.18 ± 0.24	0.19 ± 0.23	-0.00 ± 0.39	-0.01 ± 0.39	0.949
Propionivibrio sp.	0.13 ± 0.09	0.17 ± 0.11	0.20 ± 0.12	0.21 ± 0.13	0.06 ± 0.10	0.03 ± 0.14	0.655

Frigovirgula sp.	0.08 ± 0.07	0.10 ± 0.09	0.17 ± 0.21	0.09 ± 0.06	0.08 ± 0.24	-0.00 ± 0.12	0.655
D. orale	0.15 ± 0.16	0.15 ± 0.20	0.02 ± 0.02	0.02 ± 0.02	-0.12 ± 0.16	-0.13 ± 0.19	0.949
Tissierella sp.	0.01 ± 0.01	0.01 ± 0.01	0.19 ± 0.19	0.10 ± 0.11	0.17 ± 0.19	0.09 ± 0.12	0.406
